# Immune heterogeneity in cardiovascular diseases from a single-cell perspective

**DOI:** 10.3389/fcvm.2023.1057870

**Published:** 2023-04-25

**Authors:** Xin Su, Li Wang, Ning Ma, Xinyu Yang, Can Liu, Fan Yang, Jun Li, Xin Yi, Yanwei Xing

**Affiliations:** ^1^ China Academy of Chinese Medical Sciences, Guang’anmen Hospital, Beijing, China; ^2^ Department of Breast Surgery, Xingtai People’s Hospital, Xingtai, China; ^3^ Department of Breast Surgery, Dezhou Second People’s Hospital, Dezhou, China; ^4^ Fangshan Hospital Beijing University of Chinese Medicine, Beijing, China; ^5^ Department of Cardiology, Beijing Huimin Hospital, Beijing, China

**Keywords:** cardiovascular diseases, immune cell, heterogeneity, single cell RNA sequencing (scRNAseq), atherosclerosis

## Abstract

A variety of immune cell subsets occupy different niches in the cardiovascular system, causing changes in the structure and function of the heart and vascular system, and driving the progress of cardiovascular diseases (CVDs). The immune cells infiltrating the injury site are highly diverse and integrate into a broad dynamic immune network that controls the dynamic changes of CVDs. Due to technical limitations, the effects and molecular mechanisms of these dynamic immune networks on CVDs have not been fully revealed. With recent advances in single-cell technologies such as single-cell RNA sequencing, systematic interrogation of the immune cell subsets is feasible and will provide insights into the way we understand the integrative behavior of immune populations. We no longer lightly ignore the role of individual cells, especially certain highly heterogeneous or rare subpopulations. We summarize the phenotypic diversity of immune cell subsets and their significance in three CVDs of atherosclerosis, myocardial ischemia and heart failure. We believe that such a review could enhance our understanding of how immune heterogeneity drives the progression of CVDs, help to elucidate the regulatory roles of immune cell subsets in disease, and thus guide the development of new immunotherapies.

## 1. Introduction

Cardiovascular diseases (CVDs) have been recognized as the leading cause of global mortality and disability in human beings. Usual CVDs include ischemic heart disease, cardiomyopathies, arrhythmic disorders, heart failure, peripheral arterial disease, and other cardiac and vascular conditions (1, 2). Mensah et al. (3) used a vivid metaphor when explaining the global burden of cardiovascular disease in 2017: 17.8 million people died of CVDs is equivalent to reducing 330 million years of life and increasing 35.6 million years of disability. CVDs severely reduced quality of life and increased medical burden for many families. The cardiovascular system is primarily composed of cardiomyocytes, fibroblasts, endothelial cells, smooth muscle cells, pericytes, and stroma (4). Furthermore, various types of immune cells that inhabit or infiltrate cardiac tissue have been identified and characterized. All major leukocyte classes, populations of lymphocyte and myeloid origin, are present in cardiovascular tissue (5). Multiple immune cell subsets exist and occupy different niches in cardiac tissue, involving in communication with the cardiovascular system (6, 7). However, infiltrating immune cells cause structural and functional changes in the heart and vasculature, and contribute to CVDs progression (8). Targeting the inflammatory cascade in animal models has been shown to be conducive to improve myocardial injury and promote healing. In mice with deletion of interleukin 10 (IL-10), diastolic function was improved. With the occurrence of diastolic dysfunction, myocardial macrophages produce IL-10 (9). Single-cell technology allows for a more precise focus on subtypes of cells, and some research studies have confirmed that resident macrophage-derived IL-10 promotes myocardial fibrosis (10). Blocking this process could inhibit the activation of fibroblasts and reduce collagen deposition. Clinical trial has also demonstrated that inflammation is an effective therapeutic target for secondary prevention of CVDs (11). Though the study of macrophages driving CVDs is highly discussed, the great diversity and abundance of other immune cells infiltrating the damaged areas, such as a large number of lymphocytes, indicate that there is a broader dynamic immune network controlling the dynamic CVDs niche. Due to technical limitations, the effects and molecular mechanisms of these dynamic immune networks on CVDs are not adequately revealed.

Advances in single-cell applications are reshaping the way we understand the integrative behavior of immune populations, no longer inadvertently confusing or concealing the unique contributions of individual cells, especially when behavior is highly heterogeneous or driven by rare cell types (12). The high parameterization and high throughput of single-cell applications allow for in-depth characterization of low-abundance cell populations and unbiased identification (13). The use of single-cell technology to understand how immune populations use cell diversity to achieve this breadth and flexibility, especially in dynamic processes such as transdifferentiation and antigen response, provides inspiring opportunities for studying immune heterogeneity in an unprecedented scope. Given these advances, we reviewed recent work on single-cell technology, discussed data on immune heterogeneity in CVDs, and explored how immune populations generated and exploited cellular heterogeneity at multiple molecular and phenotypic levels. Furthermore, we highlighted the advantages of single-cell applications in uncovering the antecedents and consequences of immune system heterogeneity. Lastly, the latest progress toward clinical application, the remaining challenges, and future perspectives on the development of single-cell technology are briefly discussed.

## 2. Advances in single-cell applications for immune heterogeneity

The first living cells were discovered in the 17th century, but it took more than two hundred years to realize that cells are not only the structural unit of life, but also the functional unit (14). The continued exploration of cellular heterogeneity then began (15, 16). Human cells carry nearly the same genetic material, but the transcriptome information of a single cell only reflects the unique activity of a subset of genes (17). Immune cells are characterized by their heterogeneity. Studying the immune system solely at the level of cell population may not offer a comprehensive understanding, as it is crucial to explore individual immune cells' communication and masking phenotypes at the single-cell level (18). By analyzing the genomic and proteomic profiles of individual immune cells, researchers can identify previously unrecognized subpopulations and cell-to-cell interactions that contribute to the immune responses. Single-cell technology has revealed the uniqueness of individual cells and addressed questions unobtainable in bulk analysis (19, 20). Single cell RNA sequencing (scRNA-seq) analyzes the transcriptome of millions of cells at single cell resolution, achieve genome-wide characterization of the degree of transcriptional differences between coding and non-coding RNAs and generate a comprehensive gene expression atlas (21). For instance, immune cell subgroups can be disaggregated by scRNA-seq to enable characterization of heterogeneous cell populations (22, 23). This new technology can amend the developmental trajectory and classification of immune cells in the human body. Traditional hematopoietic grading is mostly based on subjectively purified cell populations that are passively segmented into component regions. Dendritic cells (DCs) differentiate, migrate and respond to environmental stimuli with a wide heterogeneity of markers and transcriptome features, suggesting that DC cells are difficult to classify precisely with predetermined markers. scRNA-seq can identify a comprehensive transcriptomic description of transient and transcriptional states. scRNA-seq introduced a paradigm shift in the classification of human DCs and identified the existence of multiple phenotypically distinct subsets in addition to the three well-defined subsets, DC1–3 (24, 25).

**Figure 1:**
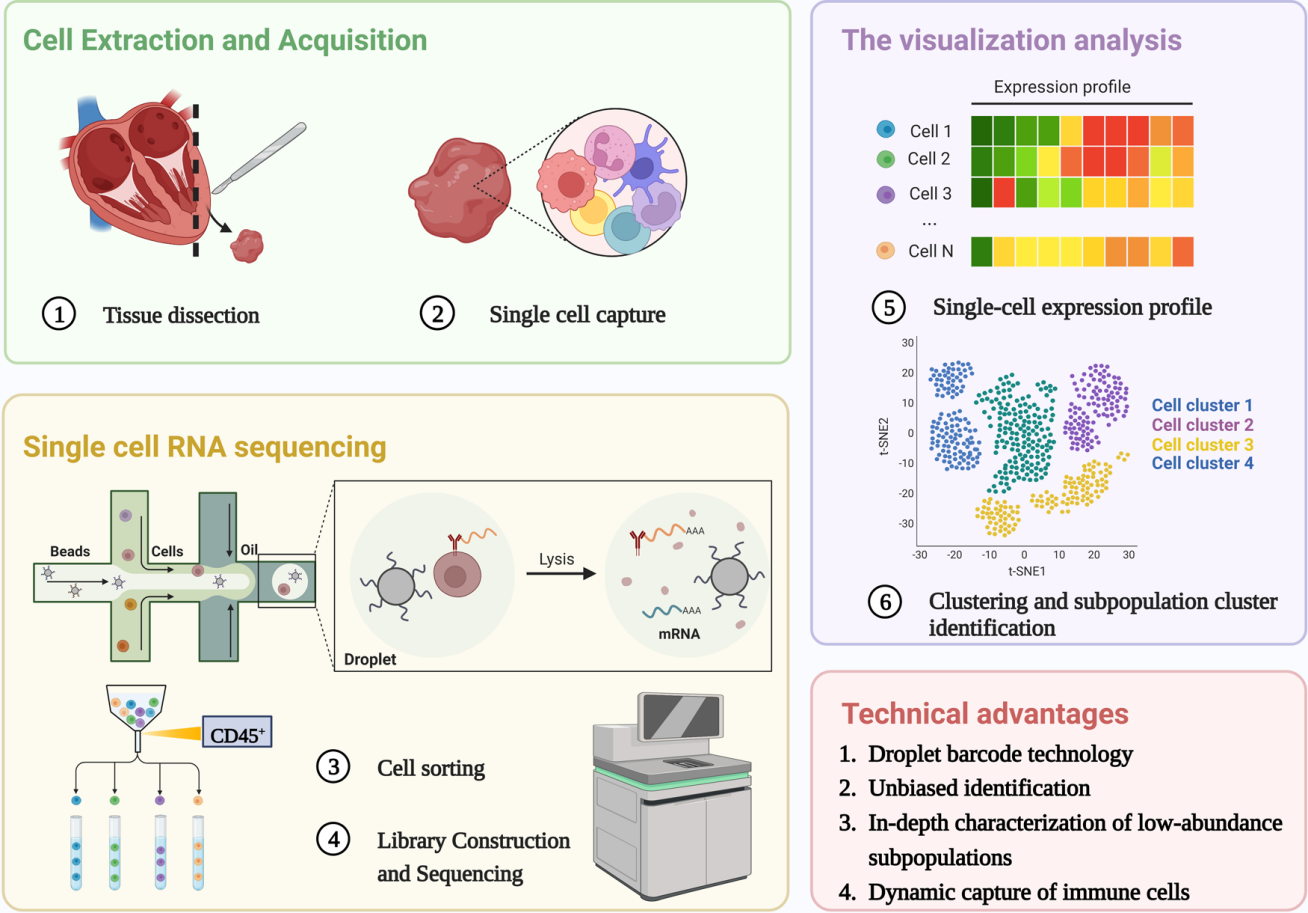
Single cell RNA sequencing workflow. The process shared by scRNA-Seq: single-cell isolation and capture, cell lysis, reverse transcription and amplification, library preparation, sequencing, and analysis. The unique advantages of scRNA seq in the study of immune heterogeneity: Application of barcode technology, unbiased identification and in-depth characterization of low abundance subpopulations, Dynamic capture of immune cells without ignoring transitional cell events. Created with BioRender.com.

scRNA-seq is reshaping the way we define the biological state of cells and is well suited to work with a limited sample size. Since the first technology was developed in 2009 (26), multiple single-cell methods have been iterated, methods for cell capture and amplification, transcript construction, and read depth per cell vary (27, 28). But in general, a similar process for scRNA-seq is: sample preparation (single-cell isolation), single-cell capture, cell lysis, reverse transcription and amplification (conversion of RNA into complementary DNA), library preparation, sequencing, and analysis (29), as shown in Figure 1. The point is to capture each individual cell in an isolated reaction mixture in which all transcripts from a single cell will be uniquely barcoded after conversion to complementary DNA (cDNA) (17). At the end of library construction, additional sample-specific barcodes are added enzymatically, allowing multiple biological samples to be sequenced simultaneously (30). This enables unique identification and access to the transcriptome of per single cell in every sample. Stoeckius et al. (31) described cellular indexing of transcriptomes and epitopes by high-throughput sequencing, a oligonucleotide-labeled antibodies phenotyping method, which is conducive to resolve distinct immune cell population with greater clarity.

The scRNA-seq data sets are high-dimensional, and analysis strategies should concentrate on highly variable genes and dimensionality reduction. Assigning biological annotations is a fundamental step that establishes the foundation for subsequent analysis of specific cell populations. In addition, functional enrichment plays a crucial role in accurately revealing the functional bias of these cell populations. Other exploratory analyses include pseudo-time analysis (32), cell-cell communication analysis (33), cell cycle prediction (34) and spatial transcriptome (35). It is noteworthy that studies on the heterogeneity of the immune system are best described using continuous models, and can only be approximated by grouping strategies that disperse cell phenotypes (13, 36). Traditional gating strategies to divide populations along any particular axis have the potential to mask the transitional cellular events that connect the complex environments. Given the asynchronous nature of the immune response, scRNA-seq generates static snapshots of the entire process which can be modeled as a coherence of transitional cellular states via capturing immune cells during a dynamic process. This kind of trajectory inference have provided a unique opportunity to track immune cells during differentiation and delineate lineage hierarchies (37). Ni et al. (38) investigated the heterogeneity of cardiac macrophages in mice with transverse aortic constriction by single-cell sequencing and identified macrophage subpopulations associated with cardiac injury. The pseudo-time trajectory confirmed that Rel gene was a key transcription factor driving CD 72^high^ macrophage differentiation which exerted a pro-inflammatory effect to induce cardiac injury. Furthermore, with the improved statistical power for large-scale single-cell datasets, it becomes possible to construct gene regulatory networks in dynamic processes by combining inferred trajectories and co-expression analyses (39). Zhuang et al. (40) analyzed the global characteristics and dynamics of single immune cells after myocardial infarction (MI), demonstrating not only dynamic inward flow of immune cells under ischemic conditions, but also identifying Fos/activator protein 1regulation as a key pro-inflammatory regulator. Multi-omics analysis further links transcriptional data with epigenetic characteristics, combines regulatory networks with clustering and trajectory inference, and constructs a regulatory landscape of the immune system (41–43). Chaffin et al. (44) formed the transcriptional landscape of the heart by analyzing left ventricular samples from dilated cardiomyopathy and hypertrophic cardiomyopathy at single-cell resolution. And They identified a reduction in the number of proliferating resident cardiac macrophages and transforming growth factor-β as the ultimate common transcriptional pathway in patients with cardiomyopathy. One such limitation is that cells of a single data set will show discrete clusters rather than continuous trajectories when dynamic trajectory analysis is performed—For example, based on the assumption that the underlying dynamic processes are stationary and ergodic, scRNA-seq captures a snapshot of cells at a particular point in time, so that scRNA-seq data with a large enough sample size can cover all possible intermediate states of a given population of cells. However, the results may not meet expectations in the case of concomitant developmental time changes or external stimuli. scRNA-seq of immune cells may aggregate according to stress-responsive genes, but is mistaken for a different cell type. Highly important but lowly expressed genes were missed due to relatively low sequencing depth in single-cell studies. In addition, bicellular cells containing 2 different cell types may be misidentified as transitional cell types.

## 3. Immune cell populations in cardiac homeostasis

Studies on the immune system have typically focused on conditions of infection/injury, but it is becoming increasingly clear that leukocytes and secreted cytokines play a role in the growth and development of tissues as well as in healthy conditions (45–47). It is well known that there are a contingent of resident immune cells in the heart, which means that immune cells are not visitors (48–50), but rather early components of heart development. Transcriptional expression of long-term tissue-resident immune cell subsets correlate with tissue site. By residing in the tissues, these immune cells are involved in tissue homeostasis and repair. We must first understand the differences in cellular composition between healthy and diseased states. The immune system plays a vital role in regulating heart health and homeostasis. Elucidating the role of each immune cell subtype and their interactions is important for understanding the mechanism of “normal-damage-repair” in the heart.

**Figure 2:**
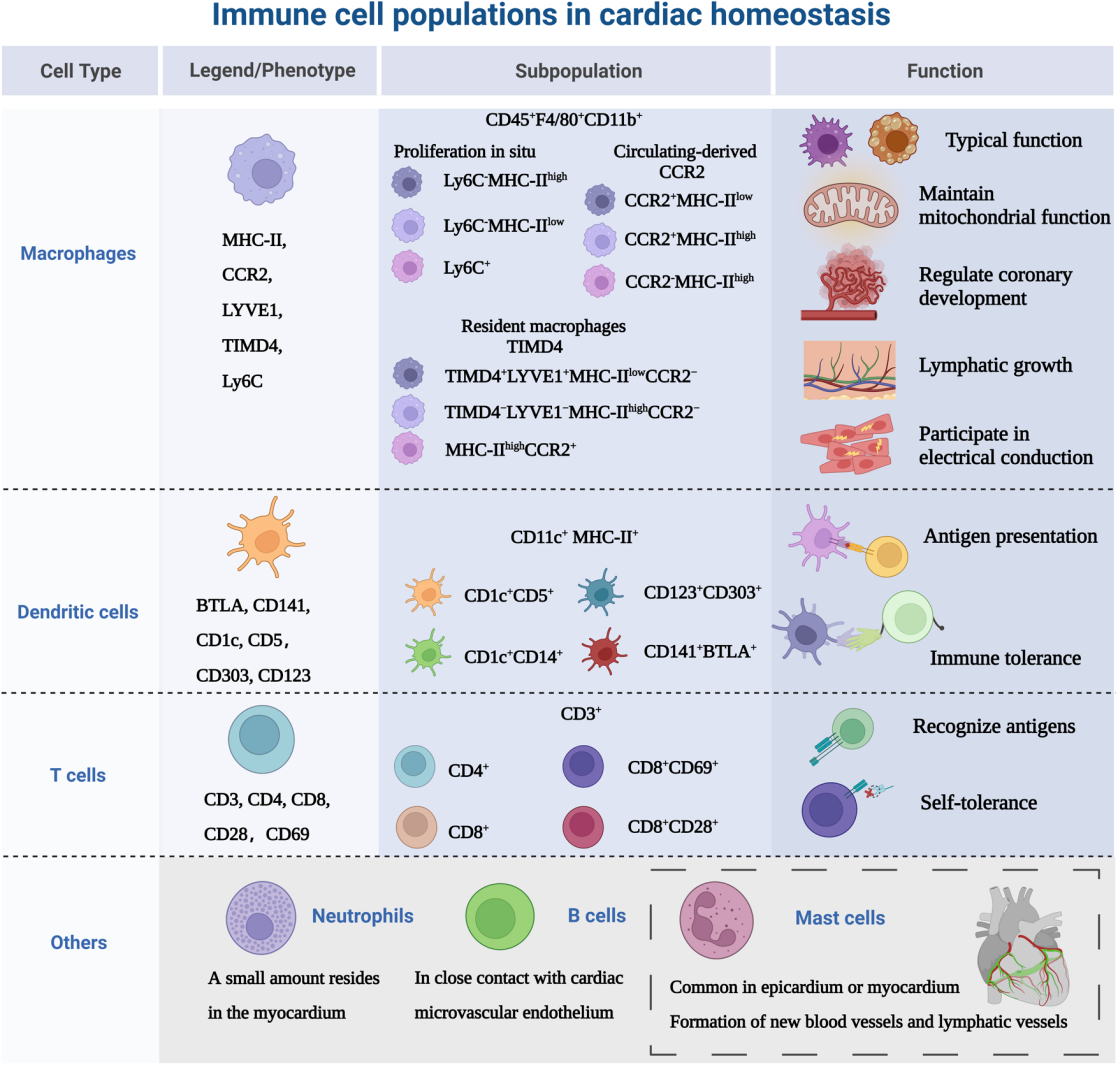
Immune cell populations in cardiac homeostasis. The study of immune cells in the absence of disease or homeostasis in the heart is equally important. Because immune cells are not “visitors” of the heart, they are the early components of the heart, not only in terms of immunity, but also involved in the growth and development of the heart. MHC-II, major histocompatibility complex class II; CCR2, CC-chemokine receptor 2; LYVE1, lymphatic vessel endothelial receptor 1; TIMD4, T cell immunoglobulin and mucin domain containing 4; Ly6C, lymphocyte antigen 6C; BTLA, B- and T-lymphocyte attenuator. Created with BioRender.com.

The heart is composed of a small population of resident immune cells from the myeloid and lymphoid lineages. The heterogeneity of those cells has proven to be overly complex to characterize, and these cell populations from multiple lineages display a wide functional diversity and dynamically express molecular markers over time. Monocytes/macrophages are representative of cardiac immune cells. Macrophages, lymphocytes, dendritic cells and a small number of neutrophils cells constitute the mainstay of cardiac immune homeostasis (51, 52). Immune cell populations sense cues from the microenvironment and respond using specific gene expression profiles. Under homeostatic/pathogenic conditions, macrophages are activated and make decisions about immune regulation in conjunction with other immune cells. In this section, we will discuss the most relevant major immune cell types, as shown in Figure 2.

### 3.1. Macrophages

Macrophage phenotype is routinely defined as CD45^+^ CD11b^+^, and murine-derived F4/80^+^ (53), but with further research, there are differences in the expression of major histocompatibility complex class II (MHC-II), CC-chemokine receptor 2 (CCR2), lymphatic vessel endothelial receptor 1 (LYVE1), T cell immunoglobulin and mucin domain containing 4 (TIMD4) and lymphocyte antigen 6C (Ly6C) in myocardium. Epelman et al. (54) found three macrophage subsets: The primary cardiac macrophage populations were Ly6C^−^MHC-II^high^, Ly6C^−^MHC-II^low^ and Ly6C^+^ which represented ∼2% of total macrophages in the adult heart. Then researchers investigated the mechanism of myocardial macrophage population recruitment in a mouse model and confirmed that these macrophages were renewed at steady state by *in situ* proliferation and were virtually unaffected by the input of circulating monocytes. Litviňuková et al. (45) used scRNA-seq to analyze the immune cell populations in adult healthy hearts and defined LYVE1^+^ TIMD4^−^-resident macrophages, speculating that these cells may be resident macrophages. It has been confirmed that the adult heart contains different resident macrophage subpopulations (55), Bajpai et al. (56) identified macrophages derived from circulating monocytes based on the expression of CCR2. The macrophages were divided into 3 distinct subsets: CCR2^+^MHC-II^low^, CCR2^+^MHC-II^high^, and CCR2^−^MHC-II^high^ cells. Meanwhile, the researchers found that in injured myocardial tissue, CCR2^+^MHC-II^low^ macrophages were only found adjacent to vessels located within areas of dense fibrosis and CCR2^−^macrophages were mostly distributed in the myocardial area absence of scar tissue. Dick et al. (57) used TIMD4 as a persistent lineage marker for resident cardiac macrophage subpopulations and observed that four macrophage subpopulations in the adult steady-state myocardium, one of which was independent of circulating monocytes (TIMD4^+^LYVE1^+^MHC-II^low^CCR2^−^), one subpopulation was partially replaced by circulating monocytes (TIMD4^−^LYVE1^−^MHC-II^high^CCR2^−^), and other two CCR2^+^MHC-II^high^ subpopulations were completely replaced by monocytes. This study illustrates the contribution of recruited monocytes to different subpopulations of resident myocardial macrophages focusing on a hierarchical discussion. Follow-up single-cell sequencing (58) further confirmed that four genes (TIMD4, LYVE1, CCR2, MHC-II) defined the macrophage subpopulations present in the heart and provided new insights into the common starting point for understanding macrophage heterogeneity. In addition to resident macrophages expressing TIMD4 and/or LYVE1, there are also MHC-II^high^ macrophages and macrophages expressing CCR2^+^. At steady state, only a modest monocyte contribution is maintained until the monocyte-derived macrophages reach a defined upper limit, which does not exceed the preexisting resident macrophages.

In addition to the typical macrophage functions, including phagocytosis and stimulation of cytokine secretion, cardiac macrophages coordinate the following functions of the heart. Primitive embryonic-derived CCR2^−^macrophages regulate coronary development through stimulation of coronary angiogenesis and remodeling of the coronary plexus (7). Driven by the cardiac autophagy mechanism, macrophages take up dysfunctional mitochondria to maintain the energy-intense unction of the heart (59). And the network of macrophages is involved in electrical conduction and lymphatic vessel growth. Hulsmans et al. (60) demonstrated that macrophages facilitated electrical conductance through the distal atrioventricular node, and that conducting cells are densely populated with elongated macrophages expressing connexin 43. In contrast, conditional deletion of connexin 43 in macrophages and lack of macrophages delayed atrioventricular conduction. Cahill et al. (61) observed that the distribution of resident macrophages in the subepicardial compartment of the developing mouse heart occurred concurrently the appearance of new lymphatics, and that macrophages interact intimately with the nascent lymphatic capillaries.

### 3.2. Dendritic cells

DCs distributed in the blood and tissues exposed to the environment, are professional antigen-presenting cells that act as a channel between the innate and adaptive immune system (62). Based on lineage, DCs are classified into three main populations: plasmacytoid (pDC) or conventional/myeloid DCs (cDC1 and cDC2) (63). There is no specific cell surface molecular marker for DCs, CD11c and MHC-II are regarded as the main markers, and they can also be expressed by macrophage subsets (64). Therefore, it is necessary to use different markers simultaneously to identify DCs. The introduction of scRNA-seq technology has greatly expanded the surface markers for identifying DCs, such as B- and T-lymphocyte attenuator (BTLA), CC-chemokine receptor 7(CCR7), CD1c, CD5 (65). DC subsets are sparsely distributed in cardiac tissue, especially abundant in the heart valves and aortic sinus, acting as “sentinels” to detect antigens (6, 66). At homeostasis, DCs share the ability of guiding immunity towards a state of tolerance (67–69). Borght et al. (70) observed in a healthy mouse model that cDC1 presented cardiac self-antigen (α-myosin heavy chain) to CD4^+^ T cells only in heart-draining mediastinal lymph nodes, resulting in Treg expansion without cardiac injury and accumulation of multiple lymphoid organs. The α-myosin heavy chain is a pathogenic autoantigen in a mouse model of spontaneous myocarditis, allowing CD4^+^ T cells to specifically evade thymic negative selection and spread to the periphery (71). In addition, proliferation and recruitment of circulating precursors regulate the turnover of cardiac cDCs (72).

### 3.3. T cells

T cells are involved in adaptive immunity by recognizing diverse antigen, maintaining immunological memory and self-tolerance (73). The main surface marker of T cells is CD3 and are divided into two subsets: CD4^+^ T cells can be divided into helper T cells (Th1, Th2 and Th17) and regulatory T cells (T_reg_). CD8^+^ T cells are called cytotoxic T cells (74). Skelly et al. (75) analyzed the transcriptional profile of non-cardiomyocytes in normal mice, and found T cells were also highly expressed Lat, Skap1, IL-17R. Unfortunately, the study did not further delineate the subtypes. It has been reported that CD69 is expressed in healthy human hearts, which may be a group of effector resident memory CD8^+^ T cells (76, 77). CD69 is expressed on activated T cells and affects Th/T_reg_ balance and suppressive activity of T_reg_, suggesting that cardiac resident T cells are associated with immune tolerance (78). To maintain immune dormancy at homeostasis, in addition to their low numbers in healthy myocardium, cardiac T cells are also regulated by self-antigen-specific tolerance (79). This process is regulated by both central and peripheral immune tolerance. Central immune tolerance refers to the clearance of immature T cells that recognize self-antigens. Self-reactive T cells that partially escape selection are inactivated by peripheral tolerance mechanisms, such as an active repression of T cell receptor signaling and a second signal, resulting in a long-term hyporesponsive state of T cells (80). Programmed cell death protein 1 (PD-1) is a T cell inhibitory molecule homologous to the co-stimulatory receptor CD28, and its role is to block activation signals from the T cell receptor and CD28 (81–83).

### 3.4. Other immune cell types involved in cardiac homeostasis

There is a gap in the knowledge of the heterogeneity of other immune cells in cardiac homeostasis. We will show the role of immune cells in the context of disease (mentioned in the next chapter). The presence of monocyte subtypes in the myocardium has been less studied with regard to monocyte/macrophage co-expressing cell subpopulations in the heart at steady state has been reported (84, 85). These subpopulations of cells are highly expressed Cd68 and Lyz2. Although extensive infiltration of neutrophils often occurs after cardiac injury (86), Martini et al. (87) used single-cell sequencing to show that small numbers of neutrophils were also present in mouse healthy myocardium. However the exact contribution of neutrophils to the heart is unknown. One study (88) confirmed that in the mouse heart, mast cells were more commonly found in the epicardium or myocardium, and scattered in the endocardium. Within the myocardium, 31% of mast cells were located around blood vessels. This is similar to the distribution of mast cells in the human heart (89). Cardiac mast cells may be involved in the formation of new blood vessels and lymphatic vessels, which are critical for cardiac development, myocardial healing, and maintenance of homeostasis (90, 91). When Adamo et al. (92) tracked myocardial B cell trafficking, they found that myocardial B cells represent a subset of circulating origin that in close contact with the cardiac microvascular endothelium, with less than 5% entered the myocardium. Analysis of B cell-deficient animals showed that cardiac B cells are involved in the growth and contractility of the myocardium (92).

## 4. The heterogeneous immune landscape in cardiovascular diseases

### 4.1. Atherosclerosis

Atherosclerosis is the underlying pathology of coronary artery disease and is regarded as a chronic inflammatory disease (93). The retained low-density lipoprotein in the arterial intima is recognized and phagocytosed by scavenger receptors on the surface of macrophages (94). This pathological process results in the accumulation of inflammatory cells and the deposition of lipid particles, which gradually form plaques in macroscopic view. Atherosclerosis is also accompanied by certain low-grade chronic inflammatory responses that attract cells of the innate and adaptive immune systems into atherosclerotic plaques.

#### 4.1.1. Inflammatory responses in atherosclerosis

He et al. (95) confirmed that the aortic arch in the aorta is prone to vascular injury. And compared with healthy mice, the immune cells of high-fat mice were more heterogeneous and pro-inflammatory cells were found: Th17 cells, CD8^+^ T cells and chemokine-enriched macrophages. The team's follow-up research found (85) that ascending aortic walls of mice fed a high-fat diet were invaded by a subset of macrophages that also amplified local inflammatory responses via scRNA-seq analysis. Four major monocyte/macrophage subpopulations were defined, including two resident clusters (cluster 1 and cluster 2), a blood-derived cluster and a B-cell-like cluster. In healthy mice, resident cluster 1 accounted for a larger proportion (>80%) in the subpopulation and significantly expressed CXC class chemokine genes. Resident cluster 2 contained proliferative properties, not only strongly expressed cell chemotactic genes and also expressed nuclear division-related genes (Top2a and Stmn1). In the presence of high cholesterol intake, the proportion of resident cluster 2 was upregulated, and cells continued to proliferate and express chemokines, increasing the likelihood that this subpopulation was programmed to exacerbate inflammation. The remaining two subpopulation had lower proportions, which might be involved in the regulation of immune effector processes. Atherosclerotic lesions are dominated by macrophages, monocytes and T cells.

The central inflammatory cells in plaques are macrophages. The classic theory of atherosclerosis holds that the classically-activated macrophages (M1)/alternatively-activated macrophages (M2) balance in plaques is dynamic, with M1 predominant in progression and M2 predominant in regression (96). Interferons induce polarization of the M1 state, and at the other end of the polarization spectrum, M2 macrophages can be induced by IL-4. Lin et al. (97) analyzed plaque cells derived from fractalkine receptor (CX3CR1)-positive precursors in mice with atherosclerosis progression or regression, revealing the transformation and activation characteristics of monocytes/macrophages. During progression of atherosclerosis, there is a more prominent heterogeneity in the activation status of macrophages, including macrophages induced by interferon and IL-4, respectively. However, there was a significant IL-4-activated macrophage population in progressive plaques compared to regressive plaques, indicating that macrophage subpopulations were not simply dichotomous. As for the specific subpopulations in regression, only a small fraction of the total cells, including a subpopulation with high expression of B cell-related genes and some subpopulations with anti-inflammatory functions. They also found a cluster of monocytes with stem-like characteristics, suggesting that circulating monocytes did not differentiate immediately after entering the myocardium, but might continue to proliferate and self-renew at sites of myocardial inflammation. These circulating mononuclear cells may play a unique role in self-repair after myocardial injury. Although macrophages in myocardial tissue are partly derived from resident macrophages, the majority of plaque macrophages are most likely derived from circulating monocytes recruited during disease progression. Cochain et al. (98) clarified the presence of CX3CR1^+^ monocyte-derived macrophage subpopulations in both the progression and regression of atherosclerosis, which represented a general inflammatory feature of atherosclerosis. These subpopulations of cells were almost exclusively detected in atherosclerotic aorta. The team described a subset of macrophages, enriched for triggered receptor expressed on myeloid cells-2 (TREM2). Such cells appear to have specialized functions in lipid metabolism and catabolism, and display gene expression signatures similar to osteoclasts, suggesting a role in pathological calcification. In addition, a class of monocyte-derived DCs with functions similar to inflammatory macrophages was defined, showing strong expression of IL-1β but low expression of chemokines. Li et al. (99) designed a program (AtheroSpectrum) to process data results from single-cell sequencing sources, revealing specific gene expression profiles associated with inflammatory macrophage foam cells, contributing to the development of therapeutic and prognostic strategies. For example, their study supported that the TREM2^+^ macrophage subpopulation was consistent with a low inflammatory profile.

Importantly, the concepts of cardiac immune heterogeneity are equally corroborated in the human heart. Fernandez et al. (100) uncovered distinct features of leukocytes in carotid artery plaques of patients. Immune cells specifically enriched in plaques included macrophages and T cells. The defined immune cells largely correspond to known immune cell populations. Plaque macrophages comprised two macrophage subsets (CD64^+^HLADR^+^CD206^high^ and CD64^+^HLADR^+^CD206^low^) based on the varied expression of the M2 marker CD206. CD8^+^ T cells and CD4^+^ T cells were enriched in the plaques. Plaque T cells display transcriptional signatures associated with cellular activation, cytotoxicity, and cellular exhaustion. CD4^+^ T cell sub-analysis showed that unlike circulating cells at rest, in plaques they displayed activated Th1 pro-inflammatory function and chemotaxis. CD8^+^ T cells in plaques not only activated the pro-inflammatory interferon-γ pathway but also activated the programmed cell death-1 signaling pathway which was a marker of T cell exhaustion (101). They found a cluster of plaque-specific T cells expressing T cell activation gene and the only cluster associated with activation of pro-inflammatory Th1 and Th17 signaling pathways. The overlapping phenomena of T cell subsets activation and depletion may be due to the progressive loss of T cell function in chronic, persistent inflammatory responses induced by unresolved plaques. T cell expression in human atherosclerotic lesions perhaps is more active. Depuydt et al. (102) applied scRNA-seq to all live cells in advanced human atherosclerotic plaques and found T cell subsets were better characterized by an activated state. CD4^+^ T cell subsets were broadly divided into cytotoxicity-signature cell cluster, regulatory T-cell cluster and central-memory cell cluster. The CD4^+^ T with cytotoxic features was shown to be characterized by the expression of PRF1 and granzyme B. In addition, a large number of T cells did not express well-defined transcription factor signals, which can be missed when analyzing human data using the classic T-helper subsets of transcription factors.CD8^+^ cell subsets were classified into cytotoxicity-signature cell cluster, effector-memory cell cluster and central-memory cell cluster as similar with CD4^+^ T cells. However, unlike the data from Fernandez et al., the researchers did not detect a clear depleted phenotype in CD8^+^ T cells.

#### 4.1.2. Chemokines and immune cells in atherosclerosis

Trajectory analysis showed that not only macrophages, but also T cells differentiated into proliferative populations when the differentiation process was activated, and the other branch was Th17 cells (103). Furthermore, macrophages influenced T cell activation by modulating MHC-II and possibly fibroblasts through the transforming growth factor β pathway. In the cellular communication analysis, T cells, fibroblasts and macrophages had significant interactions, and the most active chemotactic communication among the three was CXC chemokine ligand 12 (CXCL12)-CXC chemokine receptor 4 (CXCR4) and CC chemokine ligand 5 (CCL5)-CCR5 (103). Chemokines are a class of small molecule cytokines that induce directed chemotaxis by activating G protein-coupled receptors. Chemokine subsets are determined according to the arrangement of amino acid (N-terminal) cysteines. Most chemokines are associated with cardiovascular disease and have pro-inflammatory and leukocyte recruitment effects (104, 105). Winkels et al. (106) constructed atlas of the immune cells in mouse atherosclerosis. They found enhanced expression of inflammatory genes CXCL2, CCL2, CCL3, Ly6C2, suggesting Ly6C^+^ inflammatory monocytes. T cells clusters mainly included Th2, Th17, CD8^+^ cytotoxic T cells, memory T cells and the mix of CD4^+^/CD8^+^ T cells. Three principal B-cell subsets were detected by the markers CD43 and B220 (CD43^high^B220^neg^, CD43^neg^B220^high^, CD43^low^B220^high^). The CD43^high^B220^neg^ B cells tended to be pro-atherogenic chemokine CCL5, while CD43^neg^B220^high^ showed a predominant of the pro-inflammatory cytokine interferon γ. Further studying immune cells at different lesion sites, they found that in advanced stages of atherosclerosis, leukocyte signaling was relatively high in extravascular tissue rich in tertiary lymphoid organs, with a predominant distribution of B cells. This lesion site may be one of the major immune cell repositories in advanced atherosclerosis. CCR2, CCR5 and CX3CR1 are all involved in the recruitment of monocytes in the pathological process of atherosclerosis (107). Burger et al. (108) attempted to define macrophage subpopulations involved in atherosclerosis progression. They found that LYVE1^+^ macrophages, which express high levels of CCL24, expand under hypercholesterolemia in Apoe^−/−^ mice and promote the conversion of vascular smooth muscle cells to osteoblasts/chondrocytes in a CCL24-dependent manner. This suggests that LYVE1^+^ resident macrophages may play an important role in vascular calcification and atherosclerotic plaque instability. The process by which monocytes, guided by chemokines, enter the plaque, engulf oxidized cholesterol, convert it into foam cells, and then exit the plaque is called atherosclerosis regression. One of the features of regression is the overall reduction of plaque macrophages. Rahman et al. (109) compared plaque regression in the aortic transplantation model with normo-lipidemic recipients and those deficient in chemokine receptors, explaining the role of the above chemokines in regression. They found that recruitment of CCR2-dependent and CX3CR1-dependent monocytes was critical for the regression of plaque macrophage content. Inadequate CCR2 or CX3CR1 in aortic transplant models prevented plaque regression. Furthermore, although CCR5 promoted the progression of atherosclerosis by recruiting monocytes, it was not required for the resolution of atherosclerosis. In addition, Park et al. (110) found that C-type lectin receptor CLEC4A2 induced monocytes to join the resident macrophage pool via scRNA-seq, which had the atheroprotective property. CLEC4A is the only classical C-type lectin receptor that possesses an intracellular immunoreceptor tyrosine-based inhibitory motif, which possibly transduces negative signals (111). Through gene deletion and competitive bone marrow chimerism experiments, they determined that CLEC4A2 played a protective role in atherosclerosis by maintaining myeloid steady-state. CLEC4A2 deficiency caused the loss of homeostatic properties of resident macrophages during atherogenesis, resulting in dysfunctional cholesterol metabolism, enhanced toll-like receptor triggering and aggravating the disease.

### 4.2. Myocardial infarction

MI usually occurs when a new thrombus or ruptured plaque blocks a coronary artery. MI is a common cause of acute aseptic heart injury (112). Because the heart requires sufficient energy and oxygen to maintain continuous contraction, disruption of coronary blood supply can irreversibly kill myocardial tissue in the affected area, especially when the heart is under high load. After MI, the distribution of immune cells in myocardium has changed significantly. The plasticity of subpopulation phenotypes enhances the need to analyze the characteristics of the cellular environment by single-cell techniques. Acutely dead cardiomyocytes recruit immune cells that express different phenotypes and trigger different alarms (113).

#### 4.2.1. Intracardiac myeloid cells subpopulations

Neutrophils are short-term first responders to MI. In response to ischemic injury, circulating neutrophils and monocytes infiltrate the heart in a form that approximates a stacked wave. Calcagno et al. (114) explained the possibility of remote activation of myeloid cells from the peripheral circulation to infiltrating the infarcted heart via scRNA-seq. Similar monocyte subpopulations with high expression of interferon stimulated genes (ISGs) were found in the peripheral blood of both humans and mice suffering from MI. They also focused on myeloid cells in the infarcted heart and defined five intracardiac neutrophil subpopulations which had distinct temporal patterns between day 1 and day 4 post-MI. The first is a subpopulation of parental neutrophil that highly express Retnlg throughout the event. Another subset continued to express ISGs for 1–4 days after MI, and such expression was previously only found in monocyte-derived macrophages. The other three subpopulations highly expressed nuclear factor kappa B activation-related genes, hypoxia inducible factor 1 activation-related genes, and Siglecf, respectively. As for these three subpopulations, the first two subpopulations decreased proportionally over time and gradually were replaced by the third group. Macrophages in infarcted hearts also expressed ISGs. The team found that multiple myeloid cells at the infarct site expressed ISG and that ISG was detectable in early circulating neutrophils, which was enhanced as neutrophils matured. They also identified aberrant type I interferon innate immune activation in monocytes and neutrophils in the bone marrow of mice at steady-state and after MI. ISG became an important component in driving immune cell aggregation, suggesting that innate immunity was involved in the long-range activation associated with interferons. Macrophages have been shown to trigger a dual cascade of inflammation and repair in cardiac remodeling after MI. Farbehi et al. (115) and Jin et al. (116) successively confirmed that macrophage subpopulations have specific dominant subpopulations and dynamic changes at different stages after MI. MHC-II^high^ subsets indicate a monocytic origin and robust inflammatory potential. The researchers observed that MHC-II^high^ CCR2^high^ subsets had expanded by day 3 after MI, whereas the MHC-II^high^ CCR2^negative^ subset which shared many inflammatory features (CCL5, CXCL9, Gpb2, etc) was more noticeable at a later stage of MI (116). The subsets enriched for features related to leukocyte migration and chemotaxis was significantly increased in the acute phase (day 3) and were typical post-infarct inflammatory clusters. The next four subsets showed low levels of CCR2 and MHC-II expression with marked expansion from the restoration stage (day 7). These subsets showed the ability to resist cardiomyocyte injury, enrichment of chemokines and receptor-mediated endocytosis, and the ability to promote tissue fibrosis (angiogenesis and collagen-related gene expression), respectively (115). The profibrotic cluster manifestly appeared at day 14 after MI and involved in improving cardiac regeneration (116). Diffusion map analyses were used to model possible temporal changes in the main macrophages/monocytes population, revealing a cascade of states that break down from early infiltration and cell migration, inflammatory over-characterization, to late peak inflammatory resolution and repair.

#### 4.2.2. Lymphocyte response to myocardial infarct

In addition to the acute myeloid response, MI triggers a lymphocyte response that affects recovery. Heinrichs et al. (117) conducted in mice a phenotyping of B-cell responses in infarcted hearts to dissect the mechanisms controlling B-cell mobilization and activity *in situ*. MI triggered synchronized B-cell responses in the infarct site and the mediastinal lymph nodes. A unique B cell cluster was also identified in the infarcted heart, showing obvious chemokine receptor characteristics, with high expression of CCR7 and CXCR5. These cells were enriched in the infarct site and peaked at Day 7 after MI. Quantitative analysis found that CXCR5 ligand (CXCL13) were expressed at higher levels than CCR7 ligands (CCL19 and CCL21), and then decreased myocardial B cell infiltration after MI was observed in CXCR5-deficient mice. It is likely that healing after MI is dependent on the CXCL13- CXCR5 axis, but not CCR7, to mobilize B cells to infiltrate the myocardium.

### 4.3. Heart failure

Heart failure (HF) is primarily a clinical diagnosis that occurs secondary to either left ventricular systolic and diastolic dysfunction. Ultimately the heart is not able to transport a sufficient amount of blood under normal filling pressures to meet our body's needs. Cardiac diseases that cause HF include, but are not limited to, MI, hypertension, heart valve disease, and myocarditis (118). Myocardial injury is bound to cause aseptic myocardial inflammation; whereas termination of inflammatory infiltration requires activation of anti-inflammatory stimulus pathways, which in turn initiate profibrotic signaling (119, 120).

#### 4.3.1. Cardiac immune composition in heart failure

Under stress, immune cells even express a fibrotic phenotype (121). The relative balance between pathological inflammatory pathways and tissue repair processes determines the developmental trajectory of HF (122). Martini et al. (87) mapped the cardiac immune composition in the murine nonischemic, pressure-overload heart failure model. They demonstrated a major reorganization of immune cell abundance at late stages of HF (4 weeks after transverse aortic constriction), with macrophages and T cells being the most abundant. Activation of macrophage-dominated immune subpopulations in response to cardiac stress induced expansion, and these subpopulations clustered together macroscopically as cardiac inflammation. HF induced an increase in the abundance of resident CCR2^−^ antigen-presenting macrophages, CCR2^–^ phagocytic monocytes/macrophages, and proinflammatory, CCR2^+^Osm^+^IL-1b^+^ recruited cells to varying degrees. Oncostatin M (OSM), a member of the IL-6 family, chronic activation of which promotes the development of heart failure, functions as a major mediator of cardiomyocyte remodeling under pathological conditions (123). B cells expressed observably higher levels of CXCR5 and CCR7 after the intervention. These chemokines targeted B cells homing and directed them to the T-cell compartment of the lymph node, which increased the likelihood that B and T cells were organized into tertiary lymph nodes, as they were in atherosclerotic aortic adventitia. PD-1 was found to be present in T_reg_ cells in the early stages of the disease and promoted T_reg_-induced immunosuppressive function. T_reg_ cells were actively expressed and stable in number in the early stages of HF, and only in the later stages did they show a significant increase in number. In addition, they observed two neutrophil subpopulations, a relatively more numerous subpopulation of pro-inflammatory cells (CCR2) and a smaller subpopulation of pro-reparative cells (CCR1 and CXCR2), whose differing chemokine receptor expression might mean different microenvironment positioning modes. Koenig et al. (124) observed that HF had the greatest effect on the differential gene expression of myeloid cells and attempted to reveal their multiple subgroups. Patients with dilated cardiomyopathy displayed reduced numbers of resident macrophages and a greater number of intermediate monocytes, and other additional monocyte-derived macrophage subsets. The additional monocyte-derived macrophage subsets exhibited high chemotaxis and high inflammation. Anti-monocyte recruitment may reduce cardiac pathological inflammation and myocardial fibrosis.

#### 4.3.2. Immune cells and myocardial fibrosis in heart failure

Revelo et al. (10) analyzed the role of cardiac immune cells, especially macrophages, in the decompensated hypertrophic response of the myocardium. Macrophages played an important role in the early stages of the hypertrophic remodeling process, as evidenced by the positive correlation between the number of macrophages and the level of cardiac hypertrophy. Resident macrophages were found to be depleted early, but recruited macrophages were rapidly replenished from circulating monocytes. The macrophage-derived factors transforming growth factor-β1 and IL-10 were increased in the heart after transverse aortic constriction, and these factors had been implicated in the progression of cardiac fibrosis. The researchers found that preferential depletion of resident macrophages reduced angiogenesis and worsened fibrosis, in addition to the fact that recruited macrophages were promoters of fibrosis and stimulated the aforementioned derived factors. Resident macrophages prevent deterioration of cardiac function and ameliorate fibrosis progression after pressure overload during the early stages of cardiac remodeling. Rao's team (125) solved the gene expression disturbance between leukocytes and myocardial fibrosis. The results of the study showed that in the fibrotic myocardium, a large number of leukocytes infiltrated, especially CD8^+^ T cells and CD4^+^ T cells. In addition, tissue-resident CXCL8^high^CCR2^+^HLADR^high^ macrophages were found especially in areas of severe fibrosis. CXCL8^high^CCR2^+^HLADR^high^ cells highly express CXCL8, while its receptor duffy antigen/receptor for chemokines is expressed in activated endothelial cells. It interacted with activated endothelial cells via duffy antigen/receptor for chemokines and might promote leukocyte recruitment and infiltration in human heart failure. Several clinical studies have measured circulating subsets of monocytes or lymphocytes to assess the degree of recovery of left ventricular function or prognostic risk (126–128).

#### 4.3.3. Circulating immune cells and heart failure

Abplanalp et al. (129) analyzed the impact of circulating immune cells on HF and gave new insights into the characteristics of immune cells at the single-cell level. Although the number of monocyte subpopulations was not affected by HF, the gene expression had significantly altered. The researchers classified monocyte subpopulations according to the expression of well-established markers CD14/CD16, which were roughly divided into classical monocytes (CD14/CD16 ratio >2.5), intermediate monocytes (CD14/CD16 ratio of 0.45–2.5), and non-classical monocytes (CD14/CD16 ratio <2.5). They found specific changes in gene expression patterns in the classical monocyte subpopulations, associated with cell differentiation, regulation of cell migration and stress response. Fatty acid binding protein 5 upregulation was enriched in the classical monocytes, which contributed to the marked induction of inflammatory signatures in patients with heart failure. Intermediate monocytes were more prone to post-stress adaptive signaling, and expressions were associated with epithelial-mesenchymal transition and cell proliferation. β-catenin was deeply enriched in intermediate monocytes involved in the regulation of multiple functions, the most important of which is the “epithelial-to-mesenchymal transition”, indicating that it played an important role in HF progression and fibrosis. This suggested that modulating monocyte heterogeneity could be a new therapeutic target for rescue of HF.

## 5. Challenges and prospects

Single-cell technology uses unsupervised cluster analysis to define up to dozens of cell types, thereby assessing the importance of each cell cluster and completing the identification of key immune cell subsets. Opportunities to improve CVDs may arise following consensus on cell type and subtype identity characteristics and their functional implications. Theoretically, the selective suppression of detrimental immune cell subsets, or conversion to subsets with more beneficial functions, would lead to more effective prevention and treatment of malignant clinical events. Future increases in adaptive regulation of immune cell subpopulations, and even individualized interventions, are expected to have a profound impact on increasing life expectancy, improving quality of life, and clarifying diagnoses. Single-cell technology is now being used in clinical research. Fan et al. applied single-cell techniques to analyze the composition and phenotype of CD45+ cells in human peripheral blood samples. They revealed peripheral immune signatures associated with disease stages in patients with coronary artery disease and atherosclerotic cardiovascular disease and identified specific peripheral immune cell subpopulations that are strongly associated with coronary artery disease severity. These revealed stage-specific peripheral immune signatures become promising minimally invasive liquid biomarkers to potentially diagnose and monitor the progression of cardiovascular disease in humans (130). However, the development of such subset-specific inhibitors or reprogramming agents still needs to overcome numerous hurdles. The practical problem is that the study on immune heterogeneity cannot be separated from samples from healthy controls, and requires collecting as many immune cells as possible in the location and purifying the samples as much as possible to ensure that results are not biased towards specific cell types. Once a rare subset of immune cells has been identified using single-cell genomics, researchers need to further purify and specifically study them. The complexity of immune ecology in CVDs needs to be taken into account, and the inherent understanding of the phenotype of immune cells based on single-cell technology will be reconsidered, perhaps with the classification of gene expression profiles as a worthwhile attempt (131, 132).

As sequencing costs continue to decrease, it is economically promising to integrate multiple layers of bioinformatics and technical replication to improve the ability to detect changes in immunoheterogeneous expression. In addition, recent developments in spatial transcriptomics and multimodal omics will contribute to subpopulation-specific expression patterns and more comprehensive gene expression networks from a spatiotemporal perspective (133, 134).

## 6. Conclusion

Single-cell technology is now emerging as a powerful tool for comprehensive and unbiased analysis of the cellular composition of normal/diseased tissues. Experimental single-cell studies have revealed new heterogeneity in immune regions, demonstrating more clearly defined immune differences, compared to the relatively conserved normal situation. Single-cell techniques have also demonstrated significant immune cell cellular adaptability and plasticity in the pathological setting. In-depth single-cell studies on a limited number of precious human specimens have improved understanding of specific immune cell alterations, such as macrophages. In addition to revealing significant differences in gene expression, several variables potentially affecting immune cell behavior were analyzed in combination with in-depth analysis, considering genetic background, spatial location or intercellular interactions, showing changes in the composition of the driving cell population. The scRNA-seq analysis has remarkable resolution, even when dealing with small numbers of cells. It nicely demonstrates the enormous complexity of decision regulation in immune cells, which is a good example.

In recent years, the extensive role of immune cells in cardiovascular pathophysiology has begun to receive attention. These range from heart development and repair, to the maintenance of myocardial homeostasis, to the participation of key mechanisms of CVDs. Macrophages and T cells have the most significant heterogeneity in CVDs, their numbers and proportions are more variable than other immune cells, and they interact closely with other non-cardiomyocytes. The influence of macrophages runs through all stages of atherosclerosis, from lesion initiation and enlargement, to necrosis, and then to regression. Circulation-derived macrophages are enriched in inflammation and chemotaxis, and exhibit richer phenotypic features compared to the classical polarization classification, which may be related to their environment and activated signaling pathways. Likewise, the potent role of resident macrophages cannot be ignored, and such cells exhibit proliferative properties in the pathological process of atherosclerosis. Acute MI triggers a large number of immune cells to infiltrate the injury site, and neutrophils and monocytes overlap and accumulate at the injury site in the early response. Depletion of monocytes/macrophages affects myocardial regeneration, but the source remains to be determined (resident vs. migrating monocytes/macrophages). In addition to myeloid cells, distinct B cell subsets are defined, are strongly chemotactic and have pro-reparative effects. Immune cell heterogeneity is closely related to the time points of HF; for example, early resident macrophages have been shown to prevent cardiac deterioration, but macrophages replenished from the circulation are at risk of aggravating pathological fibrosis. Immune cell subsets are involved in signaling mechanisms regulating myocardial fibrosis, which requires further exploration of causal relationships in the future. In addition, immune cell subsets may also affect lymphatic function and remodeling, and cardiac lymph angiogenesis is also one of the therapeutic targets for HF. The appearance and gene expressions of immune cell populations at the site of injury and in circulation correlate with disease progression. As shown in Table 1, this review summarizes the phenotypic marker genes of immune cells defined through scRNA-seq. At the same time, the study of high-parameter immunophenotyping has just started, and there are many important issues to be addressed in future work. The key is to understand which immune cell subpopulations exhibit plasticity/instability and which are pathogenic clusters, and whether these can be targeted for drug therapy. Myocardial immune cell heterogeneity promotes disease progression under pathological conditions. Using healthy controls to study signature genes, combined with advances in clinical trial design, a bold approach to drug development will improve patient outcomes.

**Table 1:** Renewed phenotype marker genes of immune cells via single-cell RNA sequencing.

Disease	Model	Source of Sample	Sample Size	Methodology	Marker genes	Ref.
Under high fat condition	C57BL/6J mice	Aortic tissue	24,001	t-SNE, CellChat	Mo/MΦ cluster 1: C1q, PF4	(85)
					Mo/MΦ cluster 2: Top2a, Stmn1, Coro1a	
					Mo/MΦ cluster 3: COL1A1, Sparc, Gsn, Dcn	
					Mo/MΦ cluster 4: CD79a, CD20, Ly6d	
Under high fat/salt/glucose condition	C57BL/6J mice	Aortic tissue	216,612	t-SNE, CellPhone DB	Th17 cells: CD4, CCR6, IL-17	(95)
					CD8^+^ T cells: CD8, IFNG,	
					Macrophages: CXCL10, CXCL2, CCL3	
					B cells: CD24, IL-10	
Atherosclerosis	Ldlr^−/−^ mice	Aortic tissue	5,355	t-SNE	stem-like macrophages: CX3CR1, CD14, Adgre1, Csf1, CD68	(97)
under high fat condition	Ldlr^−/−^ mice	Aortic tissue	1,226	t-SNE, SCDE	Macrophages: TREM2	(98)
					Monocyte-derived DC/DCs: Ifi30, Napsa, CD209a	
Atherosclerosis	Human	Aortic tissue, PBMCs	9,490	CyTOF, CITE-seq, aggregated scRNA-seq	T cells: NFATC2, FYN, ZAP70, GzmA, GzmK, EOMES, PDCD1, CD223	(100)
Atherosclerosis	Human	Aortic tissue	3,282	CCA, t-SNE	CD4^+^ T cells:	(102)
					Cytotoxicity-signature cluster: GzmA, GzmK, PRF1	
					Regulatory T-cell cluster: FOXP3, CD25, CTLA4	
					Central-memory cell cluster: IL-7R, LEF1, SELL	
					CD8^+^ T cells:	
					Cytotoxicity-signature cluster: GzmB, TBX21, NKG7	
					Effector-memory cell cluster: GzmK, GzmA, CD69	
					Central-memory cell cluster: LEF1, SELL, IL-7R	
Atherosclerosis	Apoe^−/−^, Ldlr^−/−^ mice	Aortic tissue	555	PCA, t-SNE, CyTOF	T cells: CD43^high^B220^neg^, CD43^neg^B220^high^, CD43^low^B220^high^	(106)
MI	Human, C57BL/6J mice	PBMCs, heart tissue	3,102/20,206	UMAP	Neutrophils cluster 1: Retnlg	(114)
					Neutrophils cluster 2: Isg15, Ifit3, Rsad2, Ifit1, Irf7	
					Neutrophils cluster 3: Nfkb1, Icam1, IL-1a, Sod2, Tnip1	
					Neutrophils cluster 4: Egln3, Hilpda, Vegfa	
					Neutrophils cluster 5: Siglecf	
MI	C57BL/6J mice	Heart tissue	17,932	t-SNE, UMAP	Mo/MΦ cluster 1: CXCL9, Igkc, Ighm	(116)
					Mo/MΦ cluster 2: CD81, C1qa, Fcrls	
					Mo/MΦ cluster 3: Ifitm1, CD74, Napsa	
					Mo/MΦ cluster 4: CCL5, CXCL9, Gpb2	
					Mo/MΦ cluster 5: S100a9, Rsad2, Retnlg	
					Mo/MΦ cluster 6: Fabp5, Hmox1, Gpnmb	
					Mo/MΦ cluster 7: CXCL3, Sod2, Prdx1	
					Mo/MΦ cluster 8: Gas6, Cbr2, Selenop	
					Mo/MΦ cluster 9: Fn1, Slc7a2, Sdc3	
					Mo/MΦ cluster 10: Dcn, Col3a1, Bgn	
					Mo/MΦ cluster 11: Stmn1, Birc5, Top2a	
MI	C57BL/6J mice	Heart tissue	6,588	UMAP	B cells: CCR7, CXCR5, CD69	(117)
HF	C57BL/6J mice	Heart tissue	18,115	CCA, t-SNE	Mo/MΦ cluster 1: CD1, CD163, Mrc1	(87)
					Mo/MΦ cluster 2: CCR2, IL-1b, Chil3	
					Mo/MΦ cluster 3: Ear2, Eno3, CD36	
					Mo/MΦ cluster 4: CCR2, Lyz2, Ifitm6	
					B cell cluster 1: CCR7, Pax5, Ly6d	
					B cell cluster 2: CCR7, CXCR5, Mef2c	
					B cell cluster 3: CXCR5, CCR7, CD19	
					B cell cluster 4: CCR7, CD19, CX3CR1	
					CD8^+^ cell cluster 1: CD8, CD3, Thy1	
					CD8^+^ cell cluster 2: CD8, IL-17, Lat	
					CD4^+^ cell cluster 1: CD3, Lat, Lck	
					CD4^+^ cell cluster 2: Ly6c1, CD28, Tcf7	
					T_reg_ cell cluster: Foxp3, Tnfrsf18, CTL4	
					Nonexpanding T cell-like cluster: Thy1, Rora, Arg1	
					Neutrophil cluster 1: CCR2, CD69	
					Neutrophil cluster 2: CCR1, CXCR2, Arg2	
HF	Human	Heart tissue	49,723	UMAP	Macrophage cluster 1: TREM2, SPP1, LGALS3	(124)
					Macrophage cluster 2: FOLR2, LYVE1	
					Macrophage cluster 3: LYVE1, HSPH1, HSPA1A	
					Macrophage cluster 4: CCL3, CCL4, PHLDA1	
					Macrophage cluster 5: KLF2, KLF4, EGR1	
					Monocyte cluster 1: LST1, LILRA5, CD16	
					Monocyte cluster 2: FCN1, CD14, S100a8	
					Monocyte cluster 3: FCN1, OLR1, PLAUR1	

scRNA-Seq, single-cell RNA sequencing; PCA, principle component analysis; t-SNE, t-distributed stochastic neighbor embedding; Th17, T helper 17; IL, interleukin; CCR, CC-chemokine receptor; CXCL, CXC chemokine ligand; CCL, CC chemokine ligand; IFNG, interferon-gamma; Mo/MΦ, monocytes/macrophages; PF4, platelet factor 4; Top2a, topoisomerase 2alpha; STMN1, stathmin 1; Coro1a, coronin-1A; COL1A1 collagen 1A1; Sparc, secreted protein acidic and rich in cysteine; Gsn, gelsolin; Dcn, decorin; Ly6d, lymphocyte antigen 6D; SCDE, single-cell differential expression analysis;TREM2, triggered receptor expressed on myeloid cells 2; PBMCs, peripheral blood mononuclear cells; Ifi30, gamma-interferon-inducible lysosomal thiol reductase; Napsa, napsin A aspartic peptidase; Adgre1, adhesion G protein-coupled receptor E1; Csf1, colony stimulating factor 1; NFATC2, nuclear factor of activated T cells 2; FYN, Src family tyrosine kinase; ZAP70, 70-kDa zeta-associated protein; Gzm, granzyme; EOMES, eomesodermin; CCA, Canonical Correlation Analysis; PDCD1, programmed death-1; PRF1, perforin 1; FOXP3, forkhead box protein P3; CTLA432; cytotoxic T-lymphocyte-associated protein 4; LEF1, lymphoid enhancer-binding factor 1; SELL, selectin L; UMAP, uniform manifold approximation and projection; TBX21, T box 21; NKG7, natural killer cell group 7; Igkc, immunoglobulin kappa constant; Ighm, immunoglobulin M heavy chain; Fcrls, Fc receptor-like proteins; Ifitm, interferon induced transmembrane protein; Gpb2, G-protein beta 2; S100a9, S100 calcium binding protein A9; Rsad2, radical S-adenosyl methionine domain containing 2; Retnlg, resistin like gamma; Fabp5, fatty acid binding protein 5; Hmox1, heme oxygenase 1; Gpnmb, glycoprotein NMB; Sod2, superoxide dismutase 2; Prdx1, peroxiredoxin 1; Gas6, growth arrest-specific 6; Cbr2, carbonyl reductase 2; Selenop, selenoprotein P; Fn1, fibronectin1; Slc7a2, solute carrier family 7 member 2; Sdc3, syndecan 3; Col3a1, collagen type III alpha 1 chain-1; Bgn, biglycan; MI, myocardial ischemia; CLCF1, cardiotrophin-like cytokine factor 1; snRNA-Seq, single-nucleus RNA sequencing; Birc5, baculoviral IAP repeat containing 5; Areg, amphiregulin; Isg15, interferon-stimulated gene 15; Ifit3, Interferon-induced protein with tetratricopeptide repeats 3; Irf7, interferon regulatory factor 7; Nfkb1, nuclear factor of kappa light polypeptide gene enhancer in B-cells 1; Icam1, intercellular cell adhesion molecule-1; Tnip1, TNFAIP3-interacting protein 1; Egln3, egl nine homolog 3; Hilpda, hypoxia inducible lipid droplet-associated; Vegfa, vascular endothelial growth factor A; Siglecf, sialic acid-recognizing lectin F; Mrc1, C1 mannose receptor; Chil3, chitinase-like protein 3; Ear2, nuclear receptor subfamily 2 group F member 6; Eno3, enolase 3; Lyz2, lysozyme 2; Pax5, paired-box 5; Mef2c, myocyte enhancer factor 2C; Thy1, Thy-1 cell surface antigen; Lat, linker for activation of T cells; Lck, lymphocyte-specific cytoplasmic protein-tyrosine kinase p56lck; Tcf7, transcription factor 7; Tnfrsf18, tumor necrosis factor receptor superfamily member 18; Rora, RAR-related orphan receptor A; Arg1, Arginase 1; SPP1, secreted phosphoprotein 1; LGALS3, lectin, galactoside-binding, soluble 3; FOLR2, folate receptor 2; LYVE1, lymphatic vessel endothelial hyaluronan receptor 1; HSPH1, Heat shock protein 105 kDa; HSPA1A, heat shock 70 kDa; PHLDA1, pleckstrin homology-like domain, family A, member 1; KLF2, kruppel-like factor 2; EGR1, early growth response gene 1; LST1, Leukocyte Specific Transcript 1; LILRA5, leukocyte immunoglobulin-like receptor, subfamily A, member 5; FCN1, ficolin 1; OLR1, oxidized low density lipoprotein (lectin-like) receptor 1; PLAUR1, plasminogen activator, urokinase receptor 1; Apoe, apolipoprotein E; Ldlr, low-density lipoprotein receptor.

## Funding

This work was supported by the National Natural Science Foundation of China (grant number 82174349), the CACMS Innovation Fund (grant number CI2021A00919), and the National Key R&D Program of China (grant numbers 2018YFC1704901 and 2018YFC1704900).

## Conflict of interest

The authors declare that the research was conducted in the absence of any commercial or financial relationships that could be construed as a potential conflict of interest.
