# Corrigendum: Immune heterogeneity in cardiovascular diseases from a single-cell perspective

**DOI:** 10.3389/fcvm.2023.1285863

**Published:** 2023-09-15

**Authors:** Xin Su, Li Wang, Ning Ma, Xinyu Yang, Can Liu, Fan Yang, Jun Li, Xin Yi, Yanwei Xing

**Affiliations:** ^1^ China Academy of Chinese Medical Sciences, Guang’anmen Hospital, Beijing, China; ^2^ Department of Breast Surgery, Xingtai People’s Hospital, Xingtai, China; ^3^ Department of Breast Surgery, Dezhou Second People’s Hospital, Dezhou, China; ^4^ Fangshan Hospital Beijing University of Chinese Medicine, Beijing, China; ^5^ Department of Cardiology, Beijing Huimin Hospital, Beijing, China

**Keywords:** cardiovascular diseases, immune cell, heterogeneity, single cell RNA sequencing (scRNAseq), atherosclerosis

A Corrigendum on Immune heterogeneity in cardiovascular diseases from a single-cell perspective By Su X, Wang L, Ma N, Yang X, Liu C, Yang F, Li J, Yi X and Xing Y (2023) Front. Cardiovasc. Med. 10:1057870. doi: 10.3389/fcvm.2023.1057870

**Incorrect Affiliation**

In the published article, there was an error in affiliations [4,5]. Instead of “[^4^Department of Cardiology, Beijing Huimin Hospital, Beijing, China]” and “[^5^Fangshan Hospital Beijing University of Chinese Medicine, Beijing, China]”, it should be “[^4^Fangshan Hospital Beijing University of Chinese Medicine, Beijing, China]” and “[^5^Department of Cardiology, Beijing Huimin Hospital, Beijing, China]”.

The authors apologize for this error and state that this does not change the scientific conclusions of the article in any way. The original article has been updated.

